# EHS editorial 2025

**DOI:** 10.1017/ehs.2025.10034

**Published:** 2026-01-12

**Authors:** Ruth Mace

This year was a good year for our Journal. We seem to have found a niche, somewhere in the forest of new and old options open to you to publish in, for a journal that addresses the evolutionary human sciences from many different angles, ranging from primatology to human behavioural sciences. Some of our more highly read papers include those on the misuse of our disciplines, on countering scientific racism (Lala et al., 2025) and on the misuse of evolutionary psychology in the manosphere (Bachaud et al., 2025). We are publishing quite a few empirical papers on cultural evolution; I like them all but especially one on the diversification of weaving looms in Asia (Buckley et al., 2025).

We published (and have almost completed) special collections on ‘Researching Sensitive Topics’ (guest edited by Olympia Campbell and myself), which include one on gender-based violence (Campbell et al., 2025) that is currently topping our ‘most read in the last 90 days’ list; and on ‘Cultural Evolution of the Arts’ (guest edited by Oleg Sobchuk and Mason Youngblood), which ranges from Neanderthal ‘art’ (Straffon & Tennie, 2025) to video games (Valverde et al., 2025) and a lot in between. We have more collections in progress.

We welcomed a new associate editor, Katharine Balolia, underlining that palaeoanthropology is of course one of our areas of interest (and also extending our editorial geographical reach into Australia, a country that has not previously been represented on our editorial team). She and Jason Massey currently have a call out for a forthcoming special collection on ‘From primate morphology to human evolution’.

Biologically informed human sciences continue to bound ahead, partly driven by new technologies in genetics and AI. But science is also taking a few hits. The academic enterprise of human sciences, and academia in general, has less money and jobs around at the moment, partly due to political decisions (some of which are downright hostile) and partly due to global economics impacting universities. Earlier this year we lost Jane Goodall, one of the titans of an earlier era of evolutionary anthropology. If it all feels like there is a chill in the scientific weather at the moment, let’s hope it is temporary.

Suddenly, at least half of our authors now acknowledge some use of AI in creating their papers (I wonder if the question about using AI will soon become redundant). LLMs are making it easier for people to improve the flow of English in their manuscripts, as well as helping with coding, making figures, putting together datasets and many other aspects of research. I have had only one or two submissions (rejected without review) that appeared to have crossed the line into the territory of actually being ‘AI-generated’. But I do worry that these could escalate in number, potentially bringing new complications into publishing decisions in future. In our teaching, we are already grappling with how to assess the understanding our students have of our courses, given that assessment through an essay, or other coursework, is now not necessarily as reliable an indicator as it used to be.

I am now getting into the mood for Christmas, having just attended a beautiful, candle-lit carol service at my *alma mater*. In the UK, you attend religious services almost every day at school, whether or not you are religious (on my first day at school I had never heard of religion before, so was profoundly confused). Anyway, that kind of upbringing means that there is a long British tradition of celebrating our cultural history at a carol service, despite not necessarily believing in God – the music and the architecture were of course divine.

Thank you to all of you who have contributed to the journal as editors, reviewers, publishers, and authors. We need and appreciate you all. Have a great holiday, religious or otherwise. And I hope to see many of you in the historic city of Leiden, the birthplace of Rembrandt, for the EHBEA annual conference in April.
